# The ALSPAC fetal and neonatal resource: detailed data abstracted from the clinical records of the new-born

**DOI:** 10.12688/wellcomeopenres.17214.2

**Published:** 2024-03-15

**Authors:** Karen Birmingham, Yasmin Iles-Caven, Kate Northstone, Jean Golding

**Affiliations:** 1 Population Health Sciences, University of Bristol, Bristol, Avon, BS8 2BN, UK

**Keywords:** ALSPAC, Labour, Delivery, Fetus, Neonate

## Abstract

In a previous Data Note, we outlined the data obtained from clinical obstetric records concerning many details of the pregnancies resulting in the births of the children in the Avon Longitudinal Study of Parents and Children (ALSPAC). Here we describe the data that have been abstracted from medical records concerning the fetus and neonate. Full details concerning the selection biases regarding the data abstracted are outlined in the previous Data Note.

The records that have been abstracted, and described in this Data Note, concern the health of the fetus (measured in relation to the results of fetal monitoring, presentation at various stages of pregnancy, and the method of delivery) as well as the status of the newborn immediately post-delivery. Details of signs, symptoms and treatments of this population of new-born babies, as recorded in the clinical records, are described for the time during which they were in hospital or under the care of a designated midwife.

These data add depth to the information collected from elsewhere concerning this period of the child’s life: from the questionnaires completed at the time by the mother; and clinical details from neonatal intensive or special care units which will be detailed in a further Data Note.

## Abbreviations

ALSPAC    Avon Longitudinal Study of Parents and Children

CDS          Central delivery suite

CS             Caesarean section

CTG           Cardiotocography

DV            Derived variable

FSE            Fetal scalp electrode

IM            Intramuscular

IPPV         Intermittent positive pressure ventilation

IUGR         Intrauterine growth restriction

IV              Intravenous

LREC         Local Research Ethics Committee

NCE           Not classified elsewhere

N.O.S.       Not otherwise stated

NS              Not stated

OA            Occiput anterior

OP              Occiput posterior

SCBU         Special Care Baby Unit

## Introduction

The UK’s large influential National Perinatal Mortality Survey of 1958 identified fetal asphyxia as responsible for almost half of the 35 perinatal deaths per 1000 births occurring at that time ( Butler & Alberman, 1969; Butler & Bonham, 1963). By the 1990s this rate had fallen dramatically to <1 per 1000 births ( Mori *et al*., 2008). This improvement was assumed to be largely as the result of advances in the monitoring of the fetus during late pregnancy as well as more efficient methods of resuscitation. However, the consequences of these interventions (for example, inducing or augmenting labour; delivery by caesarean section [CS]; vaginal delivery using forceps or vacuum techniques; and vigorous methods of resuscitation) may have had long-term effects on the developing child. Similar questions concerning long-term effects can be asked of other problems experienced by the newborn, including the degree of jaundice, the duration of phototherapy or the exposure to antibiotics or other medications.

One of the original aims of the Avon Longitudinal Study of Parents and Children (ALSPAC) was to determine possible effects of early exposures on later health and development in childhood, adolescence and throughout adulthood ( Golding *et al*., 2001). The prevalence of many outcomes has changed over time, the extent of which has not been fully explained. These include increases in chronic childhood disorders such as diabetes (e.g., Patterson *et al*., 2012), autism spectrum disorder (e.g., Rosenberg *et al*., 2009) and obesity ( Wang & Lim, 2012) as well as in maternal prenatal depression (e.g., Pearson *et al*., 2018).

Very few longitudinal studies prior to ALSPAC had collected detailed in-depth information to allow epidemiological analyses to test whether details of fetal or neonatal exposures may be associated with these and other outcomes. Therefore, we have described here the information covering the health of the fetus that has been abstracted from obstetric records and the treatment and health of the neonate that has been abstracted from paediatric records. The data described in this Data Note differ from that in a published Data Note which describes the mother during pregnancy and the puerperium ( Birmingham *et al*., 2021a; subsequently referred to as the Obstetric Data Note). In the currrent Data Note we describe the individual data relating to the fetuses/births rather than pregnancies, thus allowing for different answers for different members of a multiple pregnancy. The data described here is therefore primarily child based). Examples concern the presentation of the fetus, fetal monitoring, and method of delivery.

## Methods

As already indicated, ALSPAC was originally designed to determine the ways in which aspects of the environment (possibly interacting with the individual’s genes) influenced child health and development ( Fraser *et al.*, 2013). The Study enrolled pregnant women who had an expected date of delivery between 1 ^st^ April 1991 and 31 ^st^ December 1992. The women had to be resident in that part of the old administrative county of Avon in south-west England comprising three District Health Authorities (Southmead, Frenchay, and Bristol and Weston) ( Boyd *et al*., 2013). Data have been collected using a variety of methods including questionnaires completed during pregnancy by the pregnant woman and her partner, abstraction of details from medical records, accurate measuring of the offspring and collection and analysis of biological samples including cord blood.

All women resident in the area at the time they were pregnant were eligible, provided that their expected date of delivery lay between 1 ^st^ April 1991 and 31 ^st^ December 1992. 14,541 pregnant women resident in the area were recruited into ALSPAC. From these pregnancies, there were a total of 14,676 fetuses and 14,062 live births. Of these children, 13,988 were still alive at one year of age. Mothers were considered enrolled if they had returned at least one questionnaire or attended a “Children in Focus” clinic by 19 ^th^ July 1999.

Most of the deliveries took place in either Southmead Hospital (53%), Bristol Maternity Hospital (now known as St Michael's Hospital) (38%) or Weston General Hospital (4%). A few deliveries took place at the mother’s home (2%) in a hospital out of the area due to the mother unexpectedly going into labour while, for example, on holiday or very occasionally when in transit to the hospital (2%).

In regard to the obstetric and neonatal records, a shortage of funding resulted in only slightly under two-thirds of the original Study sample having had data abstracted from medical records to date. The data abstraction form, abstraction instructions and checking instructions include data relating to the fetus, the newborn immediately after delivery and signs, symptoms and treatments during the first weeks of life (see *Extended data*). The choice of which records were abstracted are described in more detail in the accompanying Obstetric Data Note ( Birmingham *et al*., 2021a). That paper includes details of the likely biases incurred in analysing the data, and the possible methods of analysis. The sample includes almost all the caesarean sections, the instrumental vaginal deliveries, the pre-term deliveries, the multiple births and the fetal and neonatal deaths. There has always been the intention to complete this data extraction to include the whole of the ALSPAC enrolled population but that awaits further funding. It should be noted that extra data are available on the infants admitted to intensive or special care within the neonatal period; the detailed records are available for analysis and will be documented in a further Data Note.

### Ethics approval

An initial favourable opinion was given for ALSPAC by the three Local Research Ethics Committees (LRECs): Bristol and Weston Health Authority, (Ref E1808 28/11/1989); Southmead Health Authority, (Ref 49/89 5/04/1990); Frenchay Health Authority, (Ref 90/8 28/06/1990)]. In 1992, a general update was sent informing the LRECs of the intention to look at medical records ( Birmingham, 2018). Ethical approval was less formal at that time, with the LRECs only recently established. 

The data collected are governed by strict ethical criteria (see Birmingham, 2018) to ensure that no personal identifying information is revealed. Nevertheless, within these ethical strictures the Study encourages access to the data by *bona fide* scientists. Please note that the Study website contains details of all the data that is available through a fully searchable data dictionary and variable search tool, and a detailed proposal form for access to specified data. 

### The variable numbering system

The variable numbers for most of this data set start with either the letters ‘DEL_P’ or ‘DEL_B’ followed by a number. The distinction relates to whether the variable refers to the fetus up until the birth (DEL_P), or the baby after delivery (DEL_B). For simplicity these will be known as the P (pregnancy) and B (baby) numbers throughout this paper. In addition, the question number is quoted – i.e. the actual question asked on the data abstraction form ( *Extended data* ( Birmingham *et al*., 2021b)). 

## Data available

Due to funding restrictions, to date only 8369 pregnancies have had detailed data abstraction using the proforma shown in the data abstraction form ( *Extended data* ( Birmingham *et al.*, 2021b)). Details of the case selection and the possible biases generated are detailed in the Obstetric Data Note ( Birmingham *et al*., 2021a).

### The fetus in distress prior to delivery

***Monitoring the fetal heart rate.*** In all, there were five different types of monitoring used on the pregnant women during labour ( Table 1a), the most common being the use of a cardiotocography (CTG) monitor, continuously or intermittently. Continuous CTG monitoring does not allow the mother to move freely or change position hence the common use of intermittent CTG monitoring. A small number of records (n=38) described a different type or method of monitoring. However, for about a third of the pregnancies (n=2539) the method of monitoring was not recorded. It is likely that most of these mothers would have had intermittent CTG as the hospital protocols clearly state that normal or ‘low risk’ pregnancies should have intermittent external CTG monitoring performed on admission to the Delivery Suite and for approximately 20 minutes every 2–3 hours thereafter.

**Table 1a.** Method of monitoring the fetus.

Method	P no.	Q no.	No. with information	No. (%) using the method
CTG monitoring	1320	C14ia	7588	4022 (53%)
Continuous CTG monitoring	1321	C14ib	7558	4703 (62%)
FSE monitoring	1322	C14ic	7557	1511 (20%)
Auscultation	1323	C14id	7558	1368 (18%)
Sonicaid	1324	C14ie	7558	320 (4%)
Other type *	1325	C14ig	7558	38 (0.5%)
Unknown type	1326	C14if	7558	2539 (34%)

*Described as text, data not currently available. CTG = cardiotocography; FSE = fetal scalp electrode.

***Abnormalities of the fetal heart rate.*** Of the fetal heart rates monitored, a total of 11 different fetal heart rate patterns or abnormalities were recorded in each of the first and second stages of labour ( Table 1b, Table 1c, Table 1d). There were a small number of additional fetuses for which heart rate abnormalities were recorded but it was not clear from the records as to whether they had occurred in the first or second stage of labour (data not shown); variables have been derived to indicate whether the abnormal heart rate had occurred during labour, by combining the first and second stage occurrences with those where the timing was unknown ( Table 1d).

**Table 1b.** Fetal heart rate abnormalities in first stage of labour.

Abnormality	P no.	Q no	No. with information	No. (%) with abnormality
Any abnormality	1330	C15a1	7187	4601 (64%)
Baseline tachycardia	1331	C15a2b1	8366	351 (4%)
Tachycardia n.o.s.	1335	C15a2a1	8368	371 (4%)
Baseline bradycardia	1339	C15a2d1	8366	296 (4%)
Bradycardia n.o.s.	1343	C15a2c1	8367	830 (10%)
Type 1 dips/early decelerations	1347	C15a2e1	8360	2130 (25%)
Type 2 dips/late decelerations	1351	C15a2f1	8366	847 (10%)
Loss of beat-to-beat variability	1355	C15a2g1	8369	73 (1%)
Reduced or poor variability	1359	C15a2h1	8365	623 (7%)
Variable decelerations	1363	C15a2j1	8367	777 (9%)
Decelerations with slow recovery	1367	C15a2k1	8367	311 (4%)
Flat trace/sinusoidal pattern	1371	C15a2i1	8369	119 (1%)
Other abnormality *	1375	C15a2l1	8361	1901 (23%)

*Described as text, data not currently available. N.o.s. = not otherwise stated.

**Table 1c.** Fetal heart rate abnormalities in second stage of labour.

Abnormality	P no.	Q no	No. with information	No. (%) with abnormality
Baseline tachycardia	1332	C15a2b2	8366	239 (3%)
Tachycardia n.o.s.	1336	C15a2a2	8368	270 (3%)
Baseline bradycardia	1340	C15a2d2	8366	176 (2%)
Bradycardia n.o.s.	1344	C15a2c2	8367	841 (10%)
Type 1 dips/early decelerations	1348	C15a2e2	8360	1061 (13%)
Type 2 dips/late decelerations	1352	C15a2f2	8366	810 (10%)
Loss of beat-to-beat variability	1356	C15a2g2	8369	12 (0.1%)
Reduced or poor variability	1360	C15a2h2	8365	93 (1%)
Variable decelerations	1364	C15a2j2	8367	570 (7%)
Decelerations with slow recovery	1368	C15a2k2	8367	288 (3%)
Flat trace/sinusoidal pattern	1372	C15a2i2	8369	7 (0.1%)
Other abnormality *	1376	C15a2l2	8361	1089 (13%)

*Described as text, data not currently available. N.o.s. = not otherwise stated.

**Table 1d.** Fetal heart rate abnormalities during labour.

Abnormality	P no.	Q no	No. with information	No. (%) with abnormality
Baseline tachycardia	1334	DV	8369	591 (7%)
Tachycardia n.o.s.	1338	DV	8369	643 (8%)
Baseline bradycardia	1342	DV	8369	474 (6%)
Bradycardia n.o.s.	1346	DV	8369	1679 (20%)
Type 1 dips/early decelerations	1350	DV	8369	3193 (38%)
Type 2 dips/late decelerations	1354	DV	8369	1658 (20%)
Loss of beat-to-beat variability	1358	DV	8369	93 (1%)
Reduced or poor variability	1362	DV	8369	716 (9%)
Variable decelerations	1366	DV	8369	1351 (16%)
Decelerations with slow recovery	1370	DV	8369	599 (7%)
Flat trace/sinusoidal pattern	1374	DV	8369	126 (2%)
Other abnormality *	1378	DV	8369	3001 (36%)

*Described as text, data not currently available. N.o.s. = not otherwise stated; DV = derived variable

***Other signs of fetal compromise.*** Apart from the heart rate abnormalities, other signs of fetal distress were recorded including whether intrauterine growth restriction (IUGR) was suspected, whether meconium was seen in the liquor (and whether this was old or new), and whether an abnormal fetal blood pH had been recorded. For those with a low pH, the level is available together with the time from that level to delivery of the baby ( Table 1e).

**Table 1e.** Indications of fetal distress.

Abnormality	P no.	Q no	No. with information	No. (%) with abnormality
IUGR suspected	P1105	B666	8369	300 (4%)
Fresh meconium in liquor	P1292	C13h	8369	279 (3%)
Old meconium in liquor	P1293	C13i	8369	111 (1%)
Meconium NCE in liquor	P1294	C13j	8369	1063 (13%)
Abnormal fetal blood pH	P1391	C15b2	603	228 (3%)
- Lowest level	P1392	C15b3	226	Range 6.83-7.30
- Time lowest pH to delivery	P1393	C15b4	215	Range 1-940 min

IUGR = intrauterine growth restriction; NCE = not classified elsewhere.

### The fetus during delivery

***Position of the fetus.*** The presentation of the fetus had been recorded during pregnancy, at the start of labour and at delivery ( Table 2a). During pregnancy, the presentation was recorded on several occasions often as the result of an ultrasound examination. Consequently, there are separate variables concerning whether the baby was in a potentially problematic position prior to the onset of labour (i.e., breech, transverse lie, or an unstable lie). The actual presentation at the start of labour is denoted by one variable (vertex, breech or ‘other’ – the latter being described in text). The fifth variable indicates the actual position at the time of delivery (whether vaginal or by CS).

**Table 2a.** Presentation of the fetus during pregnancy.

Presentation	P no.	Q no.	No. with information	No. (%) involved
*During pregnancy*				
Breech	1100	B6f	8369	2927 (35%)
Transverse lie	1101	B6ff	8369	1725 (21%)
Unstable lie	1102	B6gg	8369	65 (1%)
*At onset of labour*	1200	C5a	8368	
- vertex				7614 (91%)
- breech				426 (5%)
- other *				98 (1%)
*At delivery*	1201	C5b	8149	
- vertex OA				6603 (81%)
- vertex OP				342 (4%)
- breech				344 (4%)
- other *				860 (11%)

*Described as text, data not currently available. OA = occiput anterior; OP = occiput posterior.

***Method of delivery.*** The method of delivery is described by one variable (DEL_P1210), distinguishing between assisted breech and breech extraction; assisted vaginal birth using forceps or vacuum extraction; CS, and spontaneous delivery. It should be remembered that the data are specific to the individual members of the pregnancy, each of which could have a different method of delivery. For example, there were 10 twin pregnancies in which one twin was delivered vaginally and the other by CS.

Further variables classify the types of forceps used and whether the CS was elective or emergency ( Table 2b). In order to distinguish between cases of CS that occurred after labour had started we recommend using variable P1160 which indicates whether labour had started prior to the CS; this variable is described in more detail in the companion Data Note ( Birmingham *et al.*, 2021b).

**Table 2b.** Method of delivery.

Method of Delivery	P no.	Q no.	No. with information	No. (%) involved
*Summary*	1210	C6a	8222	
Spontaneous				5025 (61%)
Assisted breech				156 (2%)
Breech extraction				6 (0.1%)
Caesarean section				1444 (18%)
Forceps				713 (9%)
Vacuum extraction				714 (9%)
Other *				164 (2%)
*Type of forceps*	1211	C6b	1134	
Wrigley’s				250 (22%)
Rhodes				443 (39%)
Neville Barnes				166 (15%)
Keilland’s				203 (18%)
Other *				72 (6%)
*Type of Caesaren section*	1212	C6c	1454	
Elective				519 (36%)
Emergency				935 (64%)

*Described as text, data are available on request. This category includes methods of delivery which had failed although a different method had succeeded (e.g. failed forceps delivery. This is the explanation for the discrepancy between the number of forceps deliveries in the first part of Table 2b and the sum of the different types of forceps used in the second part.

***The cord and placenta.*** Although the number of babies being born with their umbilical cord around their neck was relatively common (1933 births; 23%), the more dangerous cord prolapse only occurred on 24 occasions. The delivery of the placenta (or placentae in the case of most multiple births) often incurred problems of retained placenta, with consequent manual removal ( Table 2c). However, it should be noted that retained placenta was only to be coded if stated in the notes, and it was a vaginal delivery. If manual removal was recorded, the instructions were to code retained placenta – these instructions were not always followed and the appropriate edit has now been made. The actual length of the third stage of labour ranged from immediately to 275 minutes (>4 hours) as shown in Table 15 of the Obstetric Data Note ( Birmingham *et al*., 2021a).

**Table 2c.** Features concerning the umbilical cord and placenta.

Abnormality	P no.	Q no	No. with information	No. (%) with abnormality
Cord around neck	1290	C13c	8368	1933 (23%)
Cord prolapse	1291	C13d	8369	24 (0.3%)
Weight of placenta ^a^	3001	E3anot	3208	Mean 646g SD 160g
Abnormality of cord or placenta ^b^	3002	E3b	8015	1923 (24%)
Retained placenta	1402	D1p	8369	373 (4%)
Manual removal of placenta ^c^	1403	D1m	7793	360 (5%)

^a^Although the placentae were often weighed in the delivery room, there was no conformity as to whether or not the membranes or cord were included. We therefore recommend that the weight of the placenta should not be used unless measured under controlled conditions by the ALSPAC path team (see kz033); ^b^described in text, data not currently available.; ^c^in vaginal deliveries; this includes 13 multiple births where only one placenta was retained.

Most of the placentae were retained by ALSPAC if the birth took place in either of the two Bristol-based major maternity hospitals, provided the mother did not object. This involved placing the placenta immediately post-delivery into formalin in a container supplied especially for the purpose. There was rarely any standardisation at the time as to whether the membranes were retained, but a length of umbilical cord was cut and frozen at -20°C separately. Weight of the placenta was not standardised in any way and was available for only 3208 of the births in this sample. No analyses of these samples were undertaken without signed permission from the mother. Subsequent examinations of some of the placentae stored in formalin have produced measurements using a standard procedure (see Holroyd *et al*., 2016).

### The baby at birth

Outcomes of pregnancy including details of stillbirths and other deaths and measurements of the newborn will be included in a further Data Note (in preparation). See also *Treatments and procedures* below. Other baseline characteristics of the pregnancy and child are available in the ALSPAC resource with the variable names shown in Box 1.

Box 1. **Variable names for baseline characteristics of the pregnancy and child**

**Table:** 

*Feature*	*Variable*
**Multiple birth**	**Mz010a**
	
**Liveborn**	**Kz011**
**Alive at 28 days**	**Kz011a**
**Alive at 1 year**	**Kz011b**
**Sex**	**Kz021**
**Gestation**	**bestgest**
**Birthweight**	**Kz030**

***Condition at birth.*** Three different indicators of asphyxia at birth were used, including whether the baby: cried immediately; time taken before first breath (<1 minute, 1–3 minutes or >3 minutes); or was resuscitated. In addition, non-binary measurements were recorded of the time taken to establish regular respirations, and the Apgar scores at 1 and 5 minutes. Although it was relatively unusual for hospitals to record whether the baby cried immediately (26%), the other indicators were recorded in at least 85% of births ( Table 3a).

**Table 3a.** Condition of baby at birth.

Measure	B no.	Q no	No. with information	No. (%) involved
Baby cried immediately	4000	F1a	2054	1428
>3min before 1 ^st^ breath	4001	F1b	7472	59 (0.8%)
Time until regular respirations established (min)	4002	F1b	7440	Mean 1.37 SD 1.59
Apgar at 1 min	4003	F1dap1	7954	Mean 8.20 SD 1.59
Apgar at 5 min	4004	F1dap5	7951	Mean 9.44 SD 0.89
Baby was resuscitated	4005	F1e	7967	2409 (30%)

***Treatments at birth.*** Methods of resuscitation and other treatments/investigations are shown in Table 3b and Table 3c. Of the 8181individuals with detailed information 67.5% (n=5521) had no resuscitation or other treatments or investigations at, or shortly after, delivery.

**Table 3b.** Methods of resuscitation.

Method	B no.	Q No	No. with information	No. (%) using method
Bag and mask	4006	F1f1	7967	379 (5%)
Bag, mask + oxygen	4007	F1f2	7967	392 (5%)
Cardiac massage	4008	F1f3	7967	10 (0.1%)
Facial oxygen	4009	Fif4	7966	1600 (20%)
Intubation	4010	F1f5	7967	213 (3%)
IPPV+intubation	4011	F1f6	7967	226 (3%)
Mouth-to-mouth+nose	4012	F1f7	7967	0 (0%)
Ventilation n.o.s.	4013	F1f8	7967	123 (2%)
Other *	4014	F1f9	7967	152 (2%)

*Described as text, data not currently available. IPPV = intermittent positive pressure ventilation; n.o.s. = not otherwise stated.

**Table 3c.** Treatments other than resuscitation given at, or shortly after, delivery.

Treatment	B no.	Q No	No. with information	No. (%) using treatment
Naloxone	4015	F1g	7967	223 (3%)
Other drug *	4015	F1g	7967	22 (0.3%)
Suction	4016	F1h1	8182	1555 (19%)
Chest compression	4017	F1h2	8182	8 (0.1%)
Other *	4020	F1h4	8182	1284 (16%)
No treatment given	4021	F1h3	8181	5521 (67%)

*Described as text, data not currently available.

### The neonatal period

***Place and care of neonate.*** Table 4a indicates that approximately 10% of newborns were transferred immediately after delivery to a Special Care Baby Unit (SCBU) or to the transitional care ward in St Michael’s Hospital (Ward 76). This is a ward where babies can be cared for alongside their mothers who also remain in hospital. The newborns may have needed extra observations or help to feed particularly if they had been born prematurely or weighed less than 2.5 kg at birth. The duration of these admissions (if more than 24 hours) was also recorded. Those admitted to SCBU, or Neonatal Intensive Care have had detailed data collected covering their admission which will be described in a further Data Note. Nearly all the babies (98%) were discharged to their mother, with approximately 5% being re-admitted before their six-week postnatal check.

**Table 4a.** Place of care of neonate.

Process	B no.	Q no	No. with information	No. (%) involved
Transferred after delivery	4050	F2a	7982	779 (10%)
- SCBU	4050	F2a	7982	521 (7%)
- Transitional care	4050	F2a	7982	164 (2%)
- Other *	4050	F2a	7982	94 (1%)
- Duration of stay 24hr+	4051	F2c	778	741
Age at discharge (days)	4540	F12	7804	Mean 4.24 SD 7.89
Place discharged to	4550	F13a	7816 ^a^	
- Other hospital	4551	F13b		154 (2%)
- Mother				7656 (98%)
Baby readmitted before 6-week postnatal check	4600	F14	7927	363 (5%)
- Age at re-admission (days)	4601	F14	348	Mean 17.0; SD 12.0

*Described as text, data not currently available.; ^a^A further six children were either still in hospital or were discharged to others (e.g. foster parents). SCBU = Special Care Baby Unit.

The paediatric examination of the newborns included an assessment of gestation, the mean number of weeks in this cohort is estimated to be 39.5. Abnormalities of the babies’ hips were noted in just under 5% ( Table 4b).

**Table 4b.** Procedures involving the neonate.

Process	B no.	Q no	No. with information	No. (%) involved
Examined by paediatrician	7699	F7	7699	7507 (98%)
Hips examined	4350	F8a	7618	7538 (99%)
- Abnormalities noted *	4351	F8b	7537	349 (5%)
Paediatric assessment of gestation	4400	F10a	4575	Mean 39.5

*Described as text, data not currently available.

***Signs and symptoms.*** Signs and symptoms from minor to serious were noted in the babies’ first 14 days and are documented in Table 4c. The most common being jaundice (56%), ‘unsettled’ (38%) and pyrexia (28%). Other conditions that were noted were: apnoeic attacks, cyanotic attacks, high pitched or abnormal cry and sticky or moist eyes. The number of babies recorded as suffering from convulsions, umbilical infection or ‘twitching’ were in single figures only. Only 293 (4%) of the babies had no problems at all recorded.

**Table 4c.** Signs and symptoms in the neonatal period.

Sign/symptom	B no.	Q no	No. with information	No. (%) with symptom
Apnoeic attacks	4450	F11a	8135	13 (0.2%)
Cyanotic attacks	4451	F11b	8135	65 (0.8%)
Convulsions	4452	F5	7717	9 (0.1%)
High pitched/abnormal cry	4453	F11d	8135	10 (0.1%)
Twitching	4454	F11i	8135	<5 (<0.1%)
Unsettled	4455	F11k	8134	3103 (38%)
Sticky eyes	4456	F11h	8135	1612 (20%)
Moist eyes	4457	F11e	8135	1221 (15%)
Umbilical infection	4458	F11j	8135	9 (0.1%)
Mucousy	4460	F11f	8135	1769 (22%)
Jaundice	4461	F11n1	7576	4278 (56%)
- Serum bilirubin measured - - highest level (µmol) - -age (days) at highest level	4462 4463 4464	F11n2 F11n3	4265 1495 1481	1505 (35%) Mean 207 SD 62 Mean 3.8 SD 1.9
Pyrexia	4465	F11g	8134	2256 (28%)
Highest temperature	4466	F11grsit	2255	Mean 37.4 SD 0.30
Lowest temperature	4467	F6	7598	Mean 36.4 SD 0.32
Other *	4468	F11l	8134	6711 (83%)
No problems recorded	4469	F11m	8134	293 (4%)

*Described as text, data not currently available.

***Feeding and nutrition.*** As indicated in Table 4d, 69% of neonates were breast fed including those that were both breast and bottle fed with 11% recorded as having difficulties with feeding.

**Table 4d.** Feeding and nutrition.

Feeding /nutrition	B no.	Q no	No. with information	No. (%) using method
Type of feed at 24 hours	4100	F3	7923	
- Breast only				4694 (59%)
- Breast and bottle				749 (9%)
- Bottle				1728 (22%)
- Other *				752 (9%)
Feeding difficulties	4459	F11c	8135	878 (11%)

*Described as text, data not currently available.

***Vitamin K.*** Table 4e shows that Vitamin K was administered to 67% of the neonates although it is likely to have been considerably more as 2463 (32%) had no indication in the medical records that it had been administered. It is known that in one hospital, staff would document on the babies’ name cards that this vitamin had been given. These cards, which were usually attached to the mothers’ bed or babies’ cots, were frequently taken home by the mother, or discarded, but not filed in the medical records. Of those that were documented in the records, 68% were given the vitamin orally and 30% intramuscularly. 

**Table 4e.** Administration of vitamin K.

Vitamin K	B no.	Q no	No. with information	No. (%) using method
Given vitamin K	4150	F4	7663	7629
- IM				1535 (20%)
- Oral - IV - Route NS - None				3501 (46%) 130 (2%) 2463(32%) 34 (0.4%)

IM = intramuscular; IV = intravenous; NS = not stated.

Protocols from the two main hospitals state that all babies should be given the vitamin: 1mg orally for full-term normal deliveries and 0.5 mg intramuscularly for others. The hospitals differed on where the vitamin should be administered (i.e., delivery suite or ward) (see Box 2 Management Guidelines and Midwifery Operational Policies). These protocols can be found in the ALSPAC Archive in the University of Bristol Library (Special Collections Archive Box 784).

Box 2. **Management guidelines and midwifery operational policies**

**Southmead Hospital Delivery Suite**

KONAKION (PHYTOMENADIONE/VITAMIN K)

All infants should receive Vitamin K orally or IM after birth with maternal consent. The dose (0.5 - 1.0 mg) is determined by the baby’s size and mode of delivery :-

(a)      Normal delivery of full term (>37w) infant - 1 mg Vit K orally

(b)      Abnormal delivery and/or preterm (<37 weeks) infant ) - 0.5 mg Vit K, IM

and infant weighing <2.5 Kg )

VITAMIN K IS NEVER ADMINISTERED ON DELIVERY SUITE (to avoid accidental confusion with other agents e.g. oxytocic [sic]) unless mother and baby are for 6 hour discharge.

IT IS ROUTINELY GIVEN TO THE BABY ON SCBU OR ON THE POST NATAL WARDS/NURSERY AND IS INCORPORATED IN THE MIDWIVES “STANDING ORDERS”.

**Bristol Maternity Hospital Central Delivery Suite**

KONAKION (PHYTOMENADIONE/VITAMIN K)

All babies should be given Vitamin K soon after delivery. This must be recorded in the appropriate column of page 2 of the baby notes with date, time and route of administration.

All normal term infants to be given:

       
Vitamin K1 [sic] 1 mg Orally

The following babies should be given:

    
Intramuscular Vitamin K 0.5 mg

     1. Those unlikely to be fed orally in the first hours following birth, i.e.:

     a. Respiratory problems.

     b. Admissions to SCBU.

     c. Pre term (less than 36 weeks gestation).

     d. Intestinal obstruction (or other problems which may require surgery).

     e. Significant birth asphyxia.

     f. Convulsions.

     2. Those following traumatic birth or at high risk, i.e.:

     a. Breech delivery.

     b. Keillands forceps delivery.

     c. Caesarean section (other than uncomplicated elective sections at term).

     3. Other conditions i.e.:

     a. Moderate/severe Rhesus disease.

     b. Congenital infection.

     c. Bleeding disorders.

     4. Those requested by paediatricians.

Vitamin K is administered on CDS prior to transfer to wards (see Standing Order). Ward staff should be informed if Vitamin K not given for any reason.

IM = intramuscular; SCBU = Special Care Baby Unit; CDS = Central Delivery Suite. 

The article linking IM administration of Vitamin K with childhood cancer ( Golding *et al.,* 1992) was not published until August 1992 and would not have influenced the route of Vitamin K administration for this cohort as policy change was slow due to considerable controversy. However, oral administration had long been used in some Bristol units.

***Treatments and procedures.*** The neonates that had some form of treatment or investigation were 78% as can be seen in Table 4f. Although most of the types of treatment are described in text, 14% were nursed in an incubator and information on numbers using cot shields or light meters or receiving phototherapy is documented in this table also. 59% of the neonates are known to have had no drugs given while in the hospital. The mother documented the drugs given to the neonate at home in the paper questionnaire sent to her four weeks after the baby was born. These questionnaires were not sent to mothers if it was known that the baby had died or was seriously ill. Details of treatments to babies admitted to SCBU or Neonatal Intensive Care will be outlined elsewhere (in preparation). 

**Table 4f.** Treatments and procedures during the neonatal period.

Treatment or procedure	B no.	Q no	No. with information	No. (%) receiving treatment / procedure
Antibiotics given	4500	F11o1	8135	547 (7%)
Dextrose given	4501	F11o2	8135	11 (0.1%)
Other drug given *	4502	F11o3	8135	2512 (31%)
No drugs given to baby ^a^	4503	F11o4	8134	4760 (59%)
Nursed in incubator	4510	F11p3	8135	1110 (14%)
Blood sugars assessed	4512	F11p1	8135	2403 (30%)
Cot shield used	4513	F11p2	8135	490 (6%)
Light meter used	4514	F11p4	8135	175 (2%)
Meconium observations done	4515	F11p5	8135	731 (9%)
Phototherapy given	4516	F11p6	8135	216 (3%)
Other treatments *	4517	F11p7	8134	4900 (60%)
Had treatment/ investigation	4511	F11p8	8134	6318 (78%)

*Described as text, data not currently available. ^a^Known to have no drugs given but there are possibly more as some medical records were missing information on the administration of drugs.

## Strengths and limitations

There are four major strengths of these data. Firstly, each item was abstracted from the paper medical record with a strict protocol and meticulous checking; secondly, the data collected were documented at the time so that there was no element of retrospective recall; thirdly, these data can be augmented by information from the mothers’ self-completion questionnaires; and fourthly, the data provide an important baseline from which to assess the long-term benefits and possible hazards of the various facets of care.

There is one major limitation of the data - many aspects of fetal exposures, neonatal conditions and treatments are missing for over 5000 ALSPAC newborns. Admittedly, by the selection criteria used on the 8369, the majority of the more complex cases have already been abstracted, but for valid epidemiological analysis the population of all the others are also needed. It is hoped that efforts can be made in the future to fill this important gap.

## Consent

Consent to abstract data from medical records was obtained on an ‘opt out’ basis which was acceptable to the LRECs at that time. The Study Mothers were informed in the initial information booklet that their medical records would be accessed.

## Data Availability

ALSPAC data access is through a system of managed open access. The steps below highlight how to apply for access to the data included in this Data Note and all other ALSPAC data: 1. Please read the ALSPAC access policy which describes the process of accessing the data and samples in detail, and outlines the costs associated with doing so. 2. You may also find it useful to browse our fully searchable research proposal database which lists all research projects that have been approved since April 2011. 3. Please submit your research proposal for consideration by the ALSPAC Executive Committee. You will receive a response within 10 working days to advise you whether your proposal has been approved. If you have any questions about accessing data, please email alspac-data@bristol.ac.uk. The Study website also contains details of all the data that is available through a fully searchable data dictionary. Although the abstraction form, instructions and checking instructions are labelled “ALSPAC Mother during pregnancy and the pueperium”, these documents also cover data abstraction relating to the fetus, the new-born immediately after delivery and signs, symptoms and treatments during the first weeks of life. Figshare: ALSPAC Mother during pregnancy and the puerperium_data abstraction form. https://doi.org/10.6084/m9.figshare.13614701 ( Birmingham *et al.*, 2021b). Figshare: ALSPAC Mother during pregnancy and the puerperium_data abstraction instructions. https://doi.org/10.6084/m9.figshare.13621598 ( Birmingham *et al.*, 2021c). Figshare: ALSPAC Mother during pregnancy and the puerperium_ abstraction checking instructions. https://doi.org/10.6084/m9.figshare.13621703 ( Birmingham *et al.*, 2021d). Data are available under the terms of the Creative Commons Attribution 4.0 International license (CC-BY 4.0).
