# Supplementary material for: The ALSPAC fetal and neonatal resource: detailed data abstracted from the clinical records of the new-born
Source: Wellcome Open Res. 2024 Mar 15;6:298. Originally published 2021 Nov 8. [Version 2] doi: 10.12688/wellcomeopenres.17214.2 (PMC11170066; doi:10.12688/wellcomeopenres.17214.2)
Supplement: Peer review history

# Reviewer report: Reviewer response for version 2

**DOI:** 10.21956/wellcomeopenres.23494.r78444

**Published:** 2024-06-12

**Authors:** Wojciech Hanke

**Affiliations:** 1 Nofer Institute of Occupational Medicine, Łódź, Poland

The scope of information about the health of neonates presented is very extensive. However, the goal of the paper is not provided. The description of the results is very clear and the information seems valuable. The clear presentation of the goal of the paper will allow to identify better the target audience for the paper.

Are sufficient details of methods and materials provided to allow replication by others?

Yes

Is the rationale for creating the dataset(s) clearly described?

No

Are the datasets clearly presented in a useable and accessible format?

Yes

Are the protocols appropriate and is the work technically sound?

Yes

Reviewer Expertise:

Reproductive epidemiiology

I confirm that I have read this submission and believe that I have an appropriate level of expertise to confirm that it is of an acceptable scientific standard.

# Reviewer report: Reviewer response for version 2

**DOI:** 10.21956/wellcomeopenres.23494.r76930

**Published:** 2024-04-12

**Authors:** Gordon C. Smith

**Affiliations:** 1 Department of Obstetrics and Gynaecology, Cambridge University, Cambridge, UK

The authors have addressed my comments and I have no further comments to add.

Are sufficient details of methods and materials provided to allow replication by others?

Yes

Is the rationale for creating the dataset(s) clearly described?

Yes

Are the datasets clearly presented in a useable and accessible format?

Yes

Are the protocols appropriate and is the work technically sound?

Yes

Reviewer Expertise:

NA

I confirm that I have read this submission and believe that I have an appropriate level of expertise to confirm that it is of an acceptable scientific standard.

# Reviewer report: Reviewer response for version 1

**DOI:** 10.21956/wellcomeopenres.19022.r54261

**Published:** 2023-02-17

**Authors:** Neha Sethi

**Affiliations:** 1 Department of Obstetrics and Gynaecology, Faculty of Medicine, Universiti Malaya, Kuala Lumpur, Malaysia

A very well done presentation with details to the data abstraction and it's use.

 Some of the data abstracted from the medical records - example for the CTG abnormalities - does it specify if the CTG interpretation was done appropriately and followed a specific criteria? If so, it might be worth mentioning as the criteria to classify CTG and fetal heart rate changes in your data shows a consistent pattern from the records.

 In the method of delivery, with regards to forceps delivery and cesarean section, the types are mentioned differently and are different in numbers. What does it mean? The explanation is not very clear.

 The retained placenta and manual removal of placenta are mentioned as different entities. The manual removal of placenta is a procedure to remove the retained placenta. Hence why the difference. Why is it mentioned as a separate entity?

 The apgar score of baby at 5 minutes - does it only include babies who did not require any resuscitation / is there data on post resuscitative apgar scores?

 What was included in the examination of the newborn - general/systemic/biometric measurements/ponderal index/ballard score? Are there details on that?

 Is there any protocol of vaccination at birth (e.g., BCG ^1^)? If yes, it might be worth considering and including.

Are sufficient details of methods and materials provided to allow replication by others?

Yes

Is the rationale for creating the dataset(s) clearly described?

Yes

Are the datasets clearly presented in a useable and accessible format?

Yes

Are the protocols appropriate and is the work technically sound?

Yes

Reviewer Expertise:

Fetal medicine

I confirm that I have read this submission and believe that I have an appropriate level of expertise to confirm that it is of an acceptable scientific standard, however I have significant reservations, as outlined above.

## References

[1] : BCG vaccination scar associated with better childhood survival in Guinea-Bissau. *Int J Epidemiol* .2005;34(3) : 10.1093/ije/dyh392 540-7 10.1093/ije/dyh392 15659474

# Response

**Published:** 2024-03-12

**Authors:** Yasmin Iles-Caven

**Affiliations:** University of Bristol, UK

*A very well done presentation with details to the data abstraction and it's use.*

*Some of the data abstracted from the medical records - example for the CTG abnormalities - does it specify if the CTG interpretation was done appropriately and followed a specific criteria? If so, it might be worth mentioning as the criteria to classify CTG and fetal heart rate changes in your data shows a consistent pattern from the records.*

 Thank you for your comments. The data from the fetal heart rate monitoring has not been reviewed using standardised criteria. Instead we required the data transcribers to collect only the interpretations of the traces as written in the notes. We have now made this clearer in the revised version of the paper.

*In the method of delivery, with regards to forceps delivery and cesarean section, the types are mentioned differently and are different in numbers. What does it mean? The explanation is not very clear.*

 Thank you for pointing this out. We have now made the point in the text referring to this table that the data are referring to individual members of the pregnancy, not to the numbers of pregnancies. We realise that this has caused confusion among our readers and hope that by reiterating this, readers will be able to comprehend. Thus a difference in numbers between the caesarean sections can result from one twin being delivered vaginally and the other by CS. There is also a problem in interpretation of the numbers of deliveries where forceps were used. The method of delivery category ‘other’ includes methods of delivery which had failed although a different method had succeeded (e.g. failed forceps delivery is coded here rather than under the forceps category). This is the explanation for the discrepancy between the number of forceps deliveries (713) in the first part of Table 2b and the sum of the different types of forceps used in the second part (1134). We have now clarified that in the text.  

 The retained placenta and manual removal of placenta are mentioned as different entities. The manual removal of placenta is a procedure to remove the retained placenta. Hence why the difference. Why is it mentioned as a separate entity? We apologise over this confusion. There was an instruction for all cases of manual removal of the placenta to be also recorded as a retained placenta, but the edit was not undertaken. The data have been amended in the revised version of the paper.

*The apgar score of baby at 5 minutes - does it only include babies who did not require any resuscitation / is there data on post resuscitative apgar scores?*

 Apgar Scores at 1 and 5 minutes were recorded for almost all babies regardless of their resuscitation status: n= 7954 at 1 minute and n= 7954 at 5 minutes. We did not collect data on later Apgar scores.

*What was included in the examination of the newborn - general/systemic/biometric measurements/ponderal index/ballard score? Are there details on that?*

 The criteria for the examination of the newborn is likely to have varied within the different hospitals and training of the clinicians. The data abstractors were asked to only record what was written in the clinical record.

*Is there any protocol of vaccination at birth (e.g., BCG ^1^ )? If yes, it might be worth considering and including.*

 If BCG immunisation is to be given in Avon that usually happens shortly after birth, but only 1.7% of babies in this study had such an immunisation. Other immunisations were not routinely given until at least 3 months after birth. Details of which immunisations were given and when have been obtained in questionnaires completed by the study mothers at various ages and are available to researchers. We have not included the data here, but are considering developing a further data note to cover all immunisations.

# Reviewer report: Reviewer response for version 1

**DOI:** 10.21956/wellcomeopenres.19022.r53887

**Published:** 2023-01-13

**Authors:** Gordon C. Smith

**Affiliations:** 1 Department of Obstetrics and Gynaecology, Cambridge University, Cambridge, UK

Comments:

1. Page 3. The abbreviation NEC is better avoided as this is used for necrotizing enterocolitis in neonatology. Suggest use NCE = not classified elsewhere.
2. Page 3. Sentence “Here we describe the data…” I don’t really understand what they mean here.
3. Tables 1a-c. Late decelerations are thought to reflect acidosis which is quite uncommon whereas variable decelerations are thought to reflect umbilical cord compression which is relatively common. I am surprised that in all of these tables late decelerations were more common than variable decelerations. It would be good to know how this information was gleaned from the case record. Were the CTG traces reviewed and classified or were these qualities based on reading free text in the case record?
4. Table 2b. Emergency caesarean section can be further subdivided into pre-labour and intra-partum. Do they have this information?
5. Table 2c. Manual removal of placenta is only performed in the context of retained placenta. How can there by more cases of manual removal than of retained placenta? One would expect the opposite pattern as some cases of retained placenta resolve without MROP.
6. Page 8. Did the paediatric examination include basic measurements, such as head circumference and ponderal index? If so, have these data been collected?
7. Ethics and consent. I note that the study only obtained opt out consent and this was appropriate for the conduct of the study at the time. However, the data and – I assume – the biological samples are still being used now. It would be useful if they could comment more on the ethical and legal framework for sharing patient level data or storing and analysing biological samples without written consent. For example, are the biological samples held based on a Human Tissue Act license or on the basis of a current active ethics permission? Also, the offspring are now adults and it would be useful if they could explain the ethical and legal framework for storing data and samples from individuals who were infants at the time of the study but who are now adults. Alternatively, there may be other publications where these issues are covered and, if so, the authors should cite these.

Are sufficient details of methods and materials provided to allow replication by others?

Yes

Is the rationale for creating the dataset(s) clearly described?

Yes

Are the datasets clearly presented in a useable and accessible format?

Yes

Are the protocols appropriate and is the work technically sound?

Yes

Reviewer Expertise:

Epidemiological and translational research in adverse pregnancy outcome

I confirm that I have read this submission and believe that I have an appropriate level of expertise to confirm that it is of an acceptable scientific standard, however I have significant reservations, as outlined above.

# Response

**Published:** 2024-03-11

**Authors:** Yasmin Iles-Caven

**Affiliations:** University of Bristol, UK

We are very grateful for your helpful comments which we have used to improve the paper.

1. Page 3. The abbreviation NEC is better avoided as this is used for necrotizing enterocolitis in neonatology. Suggest use NCE = not classified elsewhere.

We have changed NEC to NCE as suggested throughout the paper.

1. Page 3. Sentence “Here we describe the data…” I don’t really understand what they mean here.

Apologies for the confusion. We have reworded that and the following sentence to read: ‘The data described in this Data Note differ from that in a published Data Note which describes the mother during pregnancy and the puerperium ( Birmingham *et al*., 2021a; subsequently referred to as the Obstetric Data Note). In the current Data Note we describe the individual data relating to the fetuses/births rather than pregnancies, thus allowing for different answers for different members of a multiple pregnancy. The data described here is therefore primarily child based. Examples concern the presentation of the fetus, fetal monitoring, and method of delivery.

1. Tables 1a-c. Late decelerations are thought to reflect acidosis which is quite uncommon whereas variable decelerations are thought to reflect umbilical cord compression which is relatively common. I am surprised that in all of these tables late decelerations were more common than variable decelerations. It would be good to know how this information was gleaned from the case record. Were the CTG traces reviewed and classified or were these qualities based on reading free text in the case record?

The data abstractors were expected to abstract what was recorded as free text in the clinical records and not to attempt interpretation of CTG traces. Interpretation may have been biased by the different training and skill sets among the midwives and clinicians.

  

1. Table 2b. Emergency caesarean section can be further subdivided into pre-labour and intra-partum. Do they have this information?

There is a way  of identifying CSs occurring after the onset of labour, and this is now described in the text describing Table 2b.

   5. Table 2c. Manual removal of placenta is only performed in the context of retained placenta. How can there by more cases of manual removal than of retained placenta? One would expect the opposite pattern as some cases of retained placenta resolve without MROP. Thank you for spotting this. It was due to an editing error on our part. The instructions to the data abstractors were that if a manual removal had been recorded in the clinical records, then retained placenta should automatically be coded. This had not always taken place, so a computer correction has been made. However, there was a discrepancy in 13 cases where there was a twin pregnancy – one placenta was retained, but not the other. This has now been clarified in the table and text. 

1. Page 8. Did the paediatric examination include basic measurements, such as head circumference and ponderal index? If so, have these data been collected?

Details of the outcomes of pregnancy, including birth measurements (birthweight, birth length, birth head circumference), whether live or stillborn, classification of perinatal deaths, and details of any neonatal deaths are in a further Data Note (as stated in the paper)

  

1. Ethics and consent. I note that the study only obtained opt out consent and this was appropriate for the conduct of the study at the time. However, the data and – I assume – the biological samples are still being used now. It would be useful if they could comment more on the ethical and legal framework for sharing patient level data or storing and analysing biological samples without written consent. For example, are the biological samples held based on a Human Tissue Act license or on the basis of a current active ethics permission? Also, the offspring are now adults and it would be useful if they could explain the ethical and legal framework for storing data and samples from individuals who were infants at the time of the study but who are now adults. Alternatively, there may be other publications where these issues are covered and, if so, the authors should cite these.

We have enhanced the Ethics statement to address your queries as follows: Informed consent for the use of data collected via questionnaires and clinics was obtained from participants following the recommendations of the ALSPAC Ethics and Law Committee at the time (Birmingham, 2018). All participants have the right to withdraw from the study, or elements of it, at any time – and permissions are obtained at various timepoints.  Consent for biological samples has been collected in accordance with the Human Tissue Act (2004). Where researchers have used linked data (e.g. educational and/or health records), at the age of 18, study children were sent 'fair processing' materials describing ALSPAC’s intended use of their health and administrative records and were given clear means to consent or object via a written form. Data were not extracted for participants who objected, or who were not sent fair processing materials. For linkage to their health records, ethical approval for the study was obtained from the ALSPAC Law and Ethics committee and local research ethics committees (NHS Haydock REC: 10/H1010/70). Full details of ethical approvals can be found at http://www.bristol.ac.uk/alspac/researchers/research-ethics/
